# Microglia Autophagy Mediated by TMEM166 Promotes Ischemic Stroke Secondary to Carotid Artery Stenosis

**DOI:** 10.14336/AD.2023.0803

**Published:** 2024-05-07

**Authors:** Li Li, Paul R. Krafft, Na Zeng, Ranran Duan, Xiang Qi, Anwen Shao, Fushan Xue, John H. Zhang

**Affiliations:** ^1^Department of Anesthesiology, Beijing Friendship Hospital, Capital Medical University, Beijing, China.; ^2^Department of Neurosurgery and Brain Repair, University of South Florida Morsani College of Medicine, Tampa, Florida, USA.; ^3^National Institute on Drug Dependence and Beijing Key Laboratory of Drug Dependence, Peking University, Beijing 100191, China.; ^4^Department of Neurology, The First Affiliated Hospital of Zhengzhou University, Zhengzhou, Henan, China.; ^5^Department of Anesthesiology, Sanbo Brain Hospital, Capital Medical University, Beijing, China.; ^6^Department of Neurosurgery, The Second Affiliated Hospital of Zhejiang University School of Medicine, Hangzhou, China.; ^7^Department of Anesthesiology, Neurosurgery and Neurology, School of Medicine, Loma Linda University, Loma Linda, CA, USA.; ^8^Department of Physiology and Pharmacology, Basic Sciences, School of Medicine, Loma Linda University, Loma Linda, CA, USA.

**Keywords:** Autophagy, carotid endarterectomy, microglia, stroke, TMEM166

## Abstract

Ischemic stroke can be a serious complication of selective carotid endarterectomy (CEA) in patients with carotid artery stenosis (CAS). The underlying risk factors and mechanisms of these postoperative strokes are not completely understood. Our previous study showed that TMEM166-induced neuronal autophagy is involved in the development of secondary brain injury following cerebral ischemia-reperfusion injury in rats. This current study aimed to investigate the role of TMEM166 in ischemic stroke following CEA. In the clinical part of this study, the quantitative analysis demonstrated circulating TMEM166, interleukin 6 (IL-6), and C-reactive protein (CRP) levels were significantly elevated in patients who suffered an ischemic stroke after CEA compared to those who did not. Furthermore, non-survivors exhibited higher levels of these proteins than survivors. In the preclinical part of this study, a middle cerebral artery occlusion (MCAO) model was implemented following CAS simulation in TMEM166^-/-^ mice. We found TMEM166 expression was positively correlated with the degree of ischemic brain injury. Ad5-TMEM166 transfection aggravated ischemic brain injury by inducing microglial autophagy activation and release of inflammatory cytokines. Accordingly, TMEM166 deficiency reduced brain inflammation and inhibited excessive microglial autophagy through the mammalian target of rapamycin (mTOR) pathway. These findings suggest that TMEM166 may play a key role in the development of ischemic injury after CEA and may serve as a biomarker for risk assessment of postoperative ischemic stroke.

## INTRODUCTION

TMEM166 is an endoplasmic reticulum (ER)/lysosomal protein that is widely expressed in various mammalian tissues where it plays an important role in regulating programmed cell death [1]. Although several biological functions of TMEM166 have been identified, its significance in the pathogenesis of stroke remains largely unknown. Our previous study showed that the level of TMEM166 was directly related to the degree of ischemic neuronal damage following experimental stroke in rodents [2].

Carotid artery stenosis is an essential cause and risk factor for ischemic stroke [3]. Carotid endarterectomy (CEA) is recommended for patients with high grade carotid artery disease. Although many patients benefit from CEA, approximately 2 to 20% of those who undergo surgery suffer from postoperative strokes [4]. Ischemic strokes following CEA is a complication with a low incidence but severe neurological sequelae. Although our understanding of this disease has increased over the past few years, risk factors for stroke after CEA have not yet been identified. Therefore, identifying molecular biomarkers that can accurately predict the risk of ischemic stroke after CEA could contribute to its early detection and possible treatment.

Recent studies have demonstrated that autophagy is involved in the death of various cells after stroke [5-7], including microglia [8-9]. However, the role and potential mechanisms of microglial autophagy in ischemic stroke after CEA remain unclear.

This study aims to explore the role and potential mechanisms of TMEM166 in stroke after CEA. We found that the upregulation of TMEM166 induced microglial autophagy through the mTOR signaling pathway, leading to postoperative neurological impairment in stroke patients after CEA.

## MATERIALS AND METHODS

### Participants and study approval

**Figure 1.**
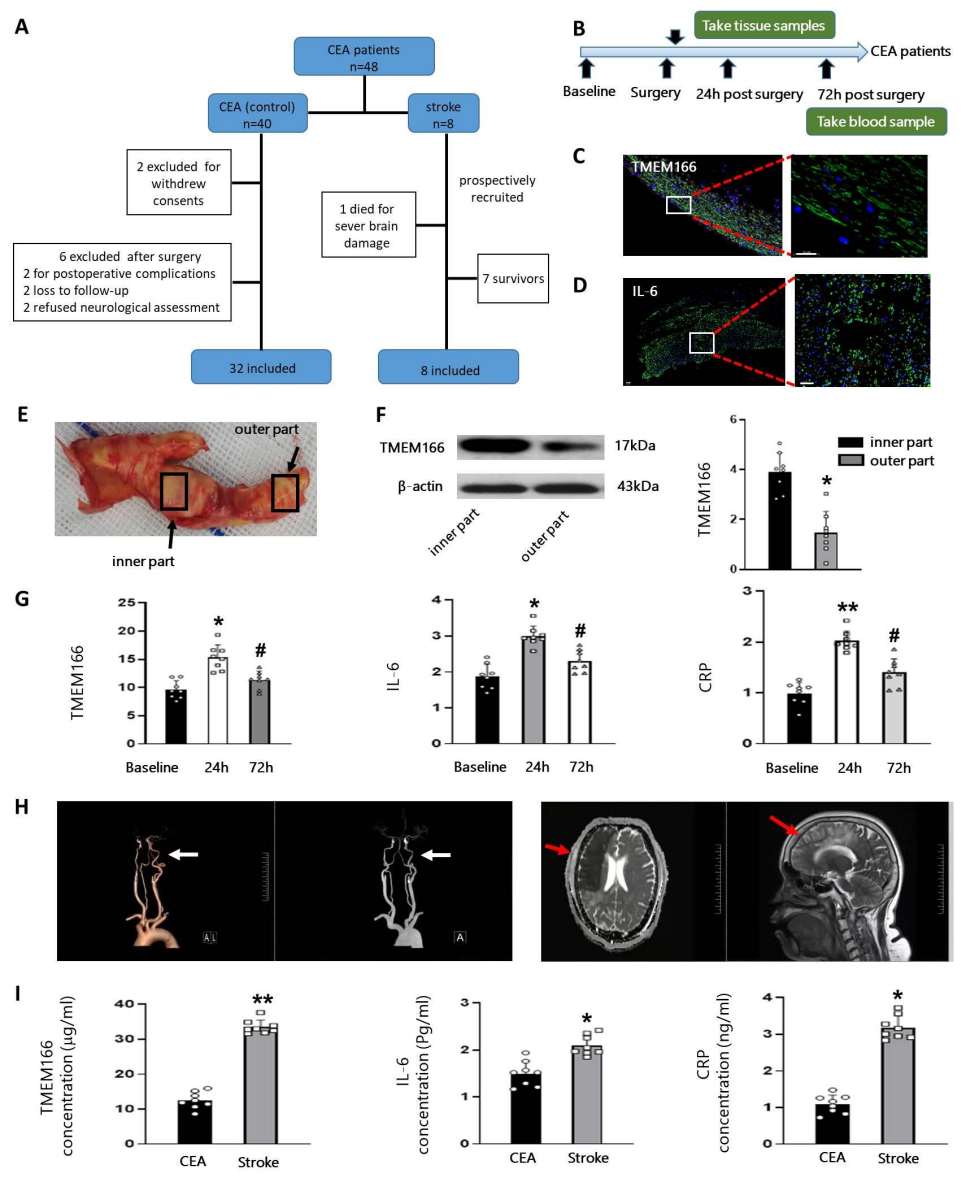
**Flowchart of the clinical study and TMEM166, cytokines expression in CEA patients**. (**A**), A detailed flowchart of participants included in the study. A total of 40 patients were included in this study, of whom 8 stroke patients were recruited after being diagnosed with new strokes following CEA, while the other 32 patients were recruited and matched in a 1:4 ratio before surgery. (**B**), The time points when blood and tissue samples were collected from patients in the CEA group are shown. (**C**) and (D), Immunofluorescence staining of TMEM166 and IL-6 in carotid artery plaques. Scale bar = 20 μm. (**E**), Carotid artery plaque obtained from carotid endarterectomy. Arrows show the thinnest and thickest parts of the tissue respectively. (**F**), The expression of TMEM166 in the thinnest and thickest parts of carotid artery plaques from CEA group patients was detected by western blot, with the average value in the thickest parts of the carotid artery plaque normalized to 1. Data are presented as mean ± SD (SD). *P < 0.05, t-test. (**G**), The relative expression levels of TMEM166, IL-6, and CRP in CEA patients were measured using quantitative PCR or ELISA. The average value in the Baseline group was normalized as 1. Data are presented as mean ± SD (SD). *P < 0.05, **P < 0.01 compared with the Baseline group, #*P* < 0.05 compared with the 24 h group, one-way ANOVA with Holm-Sidak test. (**H**), T1-weighted MRI shows a large area of infarction after CEA surgery, and CTA shows severe stenosis in the left internal carotid artery (arrowheads). These images come from the same individual. (**I**), The mean levels of TMEM166, IL-6, and CRP were compared between CEA and stroke patients. *P < 0.05, **P < 0.01, t test. *CEA* carotid endarterectomy, *MRA* magnetic resonance angiography, *CTA* computed tomography angiography.

In this study, 40 patients who underwent selective carotid endarterectomy (CEA) at Beijing Friendship Hospital of the Capital Medical University were included (July 2021 to December 2022, No. 2020-P2-301-02). Of these, 32 patients had an uncomplicated postoperative course (control group) while 8 patients experienced new strokes after CEA (stroke group). Patients who developed new comorbidities such as asthma, infections, renal or hepatic diseases within one month after surgery and those lost to follow-up were excluded (Fig. 1). All patients or their legally authorized representatives provided informed consent before participation.

### Blood and carotid plaque collection

During their hospitalization, venous blood was collected from stroke patients within 3 hours of symptom onset and then once a day. Additional blood samples were collected before as well as at 24 hours and 72 hours after CEA. The samples were centrifuged and stored at -80 °C until analysis. The levels of TMEM166, IL-6, and CRP were examined using a fluorescent quantitative PCR detection kit or ELISA kit, and analyzed using a hematology analyzer (Beckman Coulter Electronics, Fullerton, CA, USA). During CEA, carotid artery plaques were collected and fixed in formaldehyde. Following that, they were divided perpendicularly into 5 mm strips. The inner part (closest to the internal carotid artery (ICA) wall) and outer part of the specimens were obtained to evaluate TMEM166 expressions within the plaque.

### Surgery and postoperative assessment

All patients underwent CEA under general anesthesia. After successful endotracheal intubation, patients were placed in a supine position with their heads turned to the contralateral side. A longitudinal skin incision was made along the medial border of the sternocleidomastoid muscle, and the common carotid artery (CCA), external carotid artery (ECA), and ICA were exposed using a combination of blunt and sharp dissection. After temporarily clamping the ICA, CCA, and ECA, an arteriotomy of the ICA was performed and the plaque was carefully removed from the vessel wall, using microsurgical techniques. The arteriotomy was closed using 6-0 monofilament sutures. The neck incision was closed in a standard multilayered fashion and the patients were extubated and allowed to recover from general anesthesia under close observation. In this series, no patient required placement of a vascular shunt during surgery.

NIHSS score evaluation and additional radiologic evaluation including brain magnetic resonance imaging (MRI) or computed tomography angiography (CTA) were conducted on patients who developed new neurological deficits after surgery (stroke group). One month after discharge from the hospital, all patients underwent a follow-up evaluation by either telephone interview or outpatient clinic visit.

### Agents and antibodies

TMEM166 (11201ES08) Fluorescent Quantitative PCR Detection Kits were purchased from Yinsheng Biotechnology Co., Ltd. Suzhou, China. Human IL-6 (D6050) and CRP (DCRP00) Quantikine ELISA Kits and 3-MA were purchased from Cayman Chemical, USA.

The primary antibodies used in this study for immunohistochemistry and western blot experiments were as follows: anti-TMEM166 (GeneTex, 32925), transmembrane protein 119 (TMEM119) (GeneTex, 134087), Von Willebrand Factor (VWF) (Abcam, ab108918), Hoechst 33342 (Abcam, ab145597), Glial fibrillary acidic protein (GFAP) (Millipore, MAB360), NeuN (Millipore, MAB377B), β-actin (Sigma, A2066), Gapdh (Abcam, ab128915), IL-6 (Affbiotech, DF6087), IL-β (MyBioSource, MBS 125139), TNF-α (United States, 300-01A-50), IFN-γ (GeneTex, 34794), anti-LC-3 (Sigma Aldrich, L7543), mTOR (Abcam, ab32028), and phosphor-S2448 mTOR (Abcam, ab84400). Ad5-TMEM166-GFP was provided by Professor Yingyu Chen from Peking University Health Science Center in China.

Secondary antibodies for western blot and immunofluorescence included anti-rabbit IgG, HRP-linked antibody (Cell Signaling Technology, 7074s), anti-mouse IgG, HRP-linked antibody (Cell Signaling Technology, 7076s), anti-mouse DyLight 800 conjugated IgG (Rockland, 610-145-002), anti-mouse DyLight 680 conjugated IgG (Rockland, 610-144-002) or rabbit (Rockland, 611-144-002), anti-mouse IgG Alexa Fluor 594 (Thermo Fisher Scientific, A11005), and anti-rabbit IgG Alexa Fluor 488 (Thermo Fisher Scientific, A11034).

### Animals

Middle-aged male C57BL/6 mice (70-80 weeks old) were used in this study. We implemented both wild-type (WT) and TMEM166 knockout (TMEM166^-/-^) mice. All animals were housed in the Experimental Animal Department of Peking University Health Science Center and all experimental procedures were approved by the Medical Research Ethics Committee of Peking University Health Science Center and followed the "Guiding Principles for the Care and Use of Vertebrate Animals in Research and Training" of the American Physiological Society. TMEM166 knockout mice were generated by crossing offspring mice expressing TMEM166 flox alleles with Cre transgenic mice provided by the Model Animal Research Center of Nanjing University, China. Alb-Cre transgenic mice were generated by Shanghai Biomodel Organism Science and Technology Development Co., Ltd., Shanghai, China.

### Microglial Cell Culture

The mouse BV2 microglial cell line was purchased from Otwo Biotech Ltd. (Shenzhen, China), and was supplemented in dulbecco’s modified eagle medium (DMEM), maintained with 100 U/mL penicillin, 10% fetal bovine serum (FBS) and 1% penicillin. They were incubated in the control/oxygen-glucose deprivation (OGD) condition with/without the treatment of Ad5-TMEM166 at the concentration of 2 μM for 24 h. The OGD BV-2 cells were cultured in glucose-free DMEM and then maintained in a sealed incubator at 37°C 95% N_2_ and 5% CO_2_ for 4 h. The control group was cultured in normal DMEM and normoxic conditions.

### Carotid Artery Stenosis (CAS) model

The CAS mouse model was generated based on a method described by Yu Du et al. [10]. Briefly, mice were anesthetized with 1.5-2% isoflurane, and a median neck incision was made to expose the right CCA. Metal micro coils (Sawane Spring Co, Shizuoka Prefecture, Japan) with an inner diameter of 0.18 mm, an interval of 0.50 mm, and a length of 2.5 mm were wrapped around the carotid bifurcation to reduce cerebral blood flow through the right ICA. Sham-operated animals were subjected to the right CCA exposure without micro coil placement.

### Ischemic stroke model

The middle cerebral artery occlusion (MCAO) model was applied 1 month after the induction of CAS. Briefly, animals were anesthetized with isoflurane. The ICA was exposed, and the previously placed micro coils were removed. A nylon suture (RWD Life Science, MSMC23B104PK50) with silicone was inserted through the ICA stump to occlude the MCA, as described in previous studies [11-12]. During the surgery, a warming blanket was used to maintain body temperature at approximately 37°C. After 1 hour of occlusion, the suture was removed to achieve reperfusion. The animals were sacrificed, and their brains were harvested after either 24- or 72-hours following reperfusion.

### Measurement of the Cerebral Blood Flow (CBF)

Mice were anesthetized and positioned prone on a heating pad to maintain a physiologic body temperature. The skull surface was exposed by dividing the skin and periosteum, and glycerin was applied. CBF was recorded using a PeriCam PSI laser speckle imager before and after CAS, as well as at one, two, three, and four weeks after the operation. CBF was measured in a 3 x 5 mm^2^ area of the mouse brain, located between lambda and bregma, at a depth of 10 mm using the laser probe. Measurements were taken before and after the CAS simulation, as well as one, two, three, and four weeks thereafter.

### Plasmids and lentiviral vectors transfer

Ad5-TMEM166-GFP was slowly administered into the right lateral ventricle by stereotaxic injection at 2 mm posterior, 1.5 mm lateral of bregma, and 2 mm below the dura. The injection was completed using a 10 μl syringe at a rate of 0.1 μl/min, with a final volume of 3 μl of the adenovirus (2 μl of Ad5-TMEM166-GFP 1 x 10^7^ pfu/μl mixed with 1 μl of 20% mannitol in storage buffer). The glass needle was kept in the lateral ventricle for 5 minutes after injection, before being slowly removed. The skin incision was disinfected and glued using Histocryl before the mice were returned to the animal care facility. BV-2 cells were plated at the density of 2×10^5^/well in a 6-well plate and then transfected with Ad5-TMEM166 at 2 μM concentrations for an additional 24 h prior to OGD treatment.

### In vivo MRI scanning

Animals were anesthetized and immobilized using a mouse holder with bite strips and earplugs. Intravital MRI scanning was performed on a 7.0 T micromagnetic scanner (RAPID Biomedical, Rimpar, Germany) with a 23-mm diameter brain coil. T2-weighted MR images were taken 24 hours after MCAO using the following parameters: TR/TE = 400/3.5 ms, flip angle = 30°, matrix 256 x 256, and field of view 30 x 25 mm. Images were analyzed using Image J software to calculate the percentage of infarct volume.

### Western blot analysis

Western blot analyses were performed as previously described [11-12]. All mice were sacrificed after deep anesthesia, the brains were quickly removed, and the cerebral hemisphere on the injured side was dissected, frozen in liquid nitrogen, and stored at -80°C. Brain tissue and cells from each group were added to RIPA lysis buffer (sc-24948, Santa Cruz Biotechnology) with phosphatase inhibitor cocktail 2 (04693132001, Roche) and protease inhibitor PMSF (ST506, Beyotime Biotechnology) at 4°C and homogenized for 60 min. The tissue homogenate fluid was centrifuged at 14,000 g for 30 min at 4°C, and the whole cell lysate was collected. The Pierce BCA protein assay kit (23225, Thermo Fisher Scientific) was used to measure the protein concentration according to the manufacturer’s instructions, and the required loading amount (50 μg/group) was calculated and loaded on 8-15% SDS-PAGE depending on the molecular weight of the protein of interest. Proteins were transferred to nitrocellulose membranes and blocked with 5% skim milk containing Tween-20 for 2 hours at room temperature. The blocked membranes were then rinsed and incubated with primary antibodies (GFAP, β-actin, GADPH, 1:1000; TMEM119, LC3, 1:500; TMEM166, IL-6, IL-β, TNF-α, IFN-γ, mTOR, p-mTOR, 1:200) overnight at 4°C. After extensive washing with phosphate-buffered saline, 0.1% Tween-20 (TBST), the membranes were incubated with HRP-conjugated secondary antibody (1:200) for 1 hour at room temperature and visualized using the Amersham Imager 600 (General Electric Company, USA). The target bands were quantified and normalized to the control (Actin or Gapdh) using ImageJ software.

### Immunofluorescent staining

Brain tissue slices of 20-μm thickness and cell cultures were fixed in ice-cold 4% PFA, washed three times with PBS, and then permeabilized with 0.3% Triton X-100 (Aladdin, T109027) in PBS for 10 min. The slices were then blocked with 5% goat serum in PBS at room temperature for 60 min. Subsequently, the sections were incubated with primary antibodies (dilution 1:200, ) or PBS as negative control overnight at 4°C. The following day, the slices were washed thrice with PBS and incubated with secondary antibodies (dilution 1:500) for 1 hour at room temperature. After a final wash step in PBS, DAPI staining was performed (DAPI-Fluoromount-G, Southern Biotech, 0100-20) and images were captured using fluorescence microscopy (DM2500, Leica).

### Measurement of cerebral infarct volume and neurobehavioral deficits

2,3,5-triphenyl-tetrazolium sodium chloride (TTC) staining was employed to assess brain infarction volume following established protocols [2]. Briefly, mice brains were rapidly dissected, sliced into 2 mm coronal sections, and incubated in 2% TTC at 37°C for 30 minutes. Neurological deficits were assessed 24 hours post-brain injury using the modified Garcia Score, which is a sensorimotor evaluation system with a maximum score of 18. The modified Garcia test evaluates spontaneous activity, vibrissa touch, side stroking, limb symmetry, climbing, and forelimb walking, with lower scores indicating more severe injury [2]. The evaluations were performed by a blinded observer.

### Electron microscopy

Autophagy was evaluated by examining autophagic vacuoles using transmission electron microscopy. Mice were perfused with pre-cooled 0.9% NaCl followed by 4% paraformaldehyde. The parietal cortex on the injured side and the corresponding area on the contralateral side were sectioned into 0.5-mm sections, post-fixed with 1% osmium tetroxide 0.1 M calcium carbonate buffer, dehydrated in ethanol, embedded in Epon812 resin overnight, and finally stained with uranyl acetate and lead citrate. The sections were examined using a Tecnai G2 Spirit Twin 12 transmission electron microscope (Czechia) and the ultra-structures were measured using Image J software (National Institutes of Health, Bethesda, MD, USA).

### Statistical Analyses

Data were presented as mean ± standard deviation or as percentage and were tested for normality using the Shapiro-Wilks normality test. Parametric data were analyzed using one-way ANOVA, two-way ANOVA, or t-tests with GraphPad Prism 8.0.1 software. Correlation analysis was performed using Pearson's correlation test. Wilcoxon matched pairs signed rank test and paired two-tailed t-tests were used to analyze patient samples. Statistical significance was considered at *P* < 0.05. Further information on each analysis is provided in the figure legends.

## RESULTS

### Patient characteristics

A total of 48 CAS patients who underwent CEA were included in this study. Eight patients were diagnosed with new strokes after CEA, of which 1 patient with an NIHSS score > 21 died due to severe cerebral ischemia. The remaining 7 patients survived without any further stroke recurrence within 1 month (Fig. 1A). After excluding 8 CEA patients for withdrawal of consent (n = 2) or severe postoperative complications other than stroke (n = 6), the remaining patients (n = 32) were included as controls and blood samples were collected before surgery (baseline) and 24 as well as 72 hours after surgery (Fig. 1A, B). The baseline characteristics of the two groups are demonstrated in Table 1. There was no statistically significant difference between the control and the stroke group in the following basic characteristics: age (65.7 ± 1.42 vs 66.3 ± 1.76), male sex (71.9 % vs 75 %), body mass index (BMI) (24.63 ± 0.43 kg/m2 vs 25.53 ± 0.25 kg/m2), smoking habit (78.1 % vs 75 %), history of hypertension (81.3 % vs 87.5 %), coronary artery disease (14.3 % vs 12.5 %), atrial fibrillation (3.1% vs 0), diabetes mellitus (78.1 % vs 87.5 %), and regular alcohol consumption (81.3 % vs 87.5 %), respectively.

**Table 1:** Baseline characteristics of the study participants.

	Control	Stroke	
Characteristic	n = 32	n = 8	*P*
**Age (years)**	65.7 ±1.42	66.3 ±1.76	0.35
**Male sex, n (%)**	23 (71.9)	6 (75)	0.42
**BMI (kg/m^2^)**	24.63 ± 0.43	25.53 ± 0.25	0.36
**Smoking, n (%)**	25 (78.1)	6 (75)	0.63
**Hypertension, n (%)**	26 (81.3)	7 (87.5)	0.75
**Coronary heart disease, n (%)**	5 (14.3)	1 (12.5)	0.9
**Atrial fibrillation, n (%)**	1 (3.1)	0	0.89
**Diabetes mellitus, n (%)**	25 (78.1)	7 (87.5)	0.52
**Drinking, n (%)**	26 (81.3)	7 (87.5)	0.67
**Total cholesterol (mmol/L)**	4.06 ± 0.49	4.13 ± 0.42	0.89
**Triglycerides (mmol/L)**	1.54 ± 0.16	1.85 ± 0.16	0.92
**LDL (mmol/L)**	3.49 ± 0.43	3.60 ± 0.12	0.91
**HDL (mmol/L)**	1.23 ± 0.12	1.32 ± 0.12	0.79
**Previous contralateral CEA**	2 (6.25)	1 (12.5)	0.42
**Duration of surgery (min)**	147 ± 26.8	157 ± 15	0.59
**Midazolam (mg/kg)**	0.047 ± 0.002	0.047 ± 0.025	0.94
**Fentanyl (ug/kg)**	1.7 ± 0.18	1.7 ± 0.53	0.95
**TMEM166 (ug/mL) average**	12.54 ± 3.74	38.01 ± 10.01	< 0.01
**IL-6 (pg/mL) average**	1.02 ± 0.27	3.08 ± 0.78	< 0.05
**CRP (ng/mL) average**	1.64 ± 0.60	3.09 ± 1.38	< 0.05
**Side of operation (left/right)**	27.5%/72.5%	12.5%/87.5%	0.54
**NIHSS score**			
**(1-4)**		1 (62.5)	
**(5-15)**		4 (12.5)	
**(15-20)**		2 (12.5)	
**(21-42)**		1 (12.5)	

**Figure 2.**
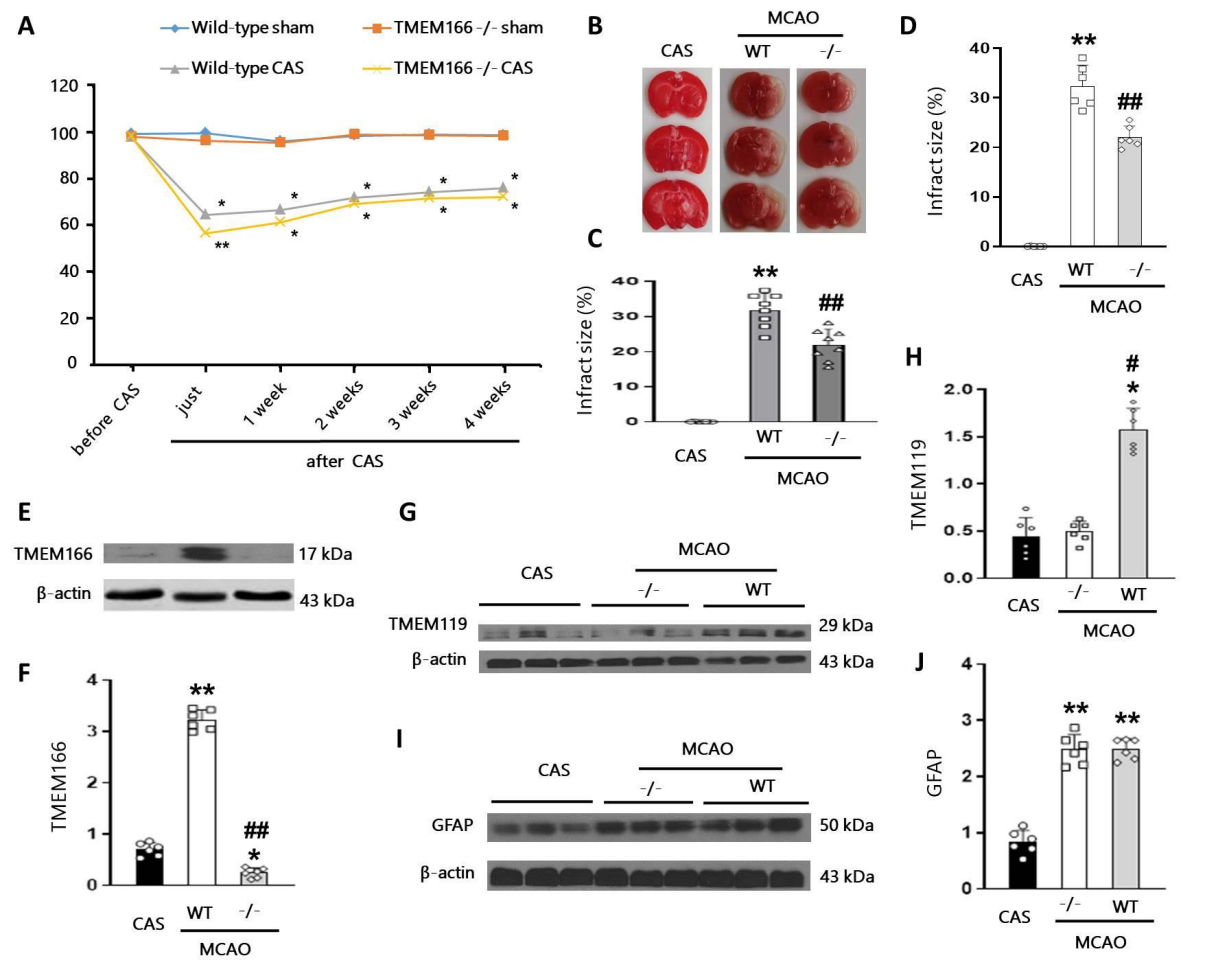
**Post-CEA brain ischemic model establishment and protein levels of TMEM166 and TMEM119 24 hours after ischemic stroke secondary to CAS**. (**A**), Cerebral blood flow (CBF) changes in different groups of mice. n = 8 per group. Data are presented as mean ± SD (SD). *P < 0.05, **P < 0.01 compared with the sham group, one-way repeated measures ANOVA with Holm-Sidak test. (**B-C**), Representative images of TTC stained sections and the quantification of infarct volume in different groups. n = 8 per group. Data are presented as mean ± SD (SD). **P < 0.01 compared with the CAS group, ^##^*P* < 0.01 compared with MCAO+WT group, one-way ANOVA with Holm-Sidak test. (**D-F**), The quantification of infarct volume in different groups using T2 weighted MR images and the expression of TMEM166 was measured by western blot. n = 6 per group. **P < 0.01 compared with the CAS group, ^##^*P* < 0.01 compared with MCAO+WT group, one-way ANOVA with Holm-Sidak test. (**G-J**), The expression of TMEM119 and GFAP was measured by western blot. n = 6 per group. Data are presented as mean ± SD (SD). *P < 0.05, **P < 0.01 compared with the CAS group, ^#^*P* < 0.05 compared with MCAO+TMEM166^-/-^ group, one-way ANOVA with Holm-Sidak test. *WT*, wild type.

Furthermore, patients in both groups had similar lipid profiles, the incidence of previous contralateral CEA, as well as surgical parameters such as operative time, side of the operation, and dose of anesthetics. Serum levels of TMEM166 (12.54 ± 3.74 ug/mL vs 38.01 ± 10.01 ug/mL, p<0.01), IL-6 (1.02 ± 0.27 pg/mL vs 3.08 ± 0.78 pg/mL, p<0.05) and CRP (1.64 ± 0.6 ng/mL vs 3.09 ± 1.38 ng/mL, p<0.05) after CEA in perioperative stroke patients were statistically higher than in patients within the control group.

All patients with neurological symptoms after CEA were scored using the NIHSS (Table 1). One patient with a high score passed away, and all patients in the control group were discharged in good health without any adverse events.

### TMEM166 and inflammatory cytokines were increased in stroke patients after CEA

The inner and outer parts of the sclerotic plaque (tissue close to the normal internal carotid artery wall) were separated to evaluate the expression of TMEM166 in CEA patients. Immunofluorescence staining (Fig. 1C) and western blot (Fig. 1E, F) results suggested that TMEM166 was abundantly expressed in carotid artery plaque specimens, especially in the thickest part of the plaque. IL-6 and CRP are widely studied inflammatory markers and have been found to be associated with atherosclerosis plaque development, especially in elderly stroke and transient cerebral ischemia patients [13-14]. In this study, immunofluorescence staining also detected localized IL-6-positive immune cells within the plaques (Fig. 1D). Serum levels of TMEM166, IL-6, and CRP were measured in CEA patients. Concentrations of all those markers were significantly increased 24 hours after surgery, which decreased within 72 hours (Fig. 1G). These results suggest that the high expression of TMEM166 in CAS may be related to inflammatory pathways.

Of the 8 patients admitted to the intensive care unit with ischemic stroke after CEA, one patient developed a large brain infarction after surgery and passed away on the 4^th^ day after hospitalization (Fig. 1H). This patient was found to have a higher TMEM166 serum level (51.87 ug/mL) than those patients who survived post-CEA strokes (n = 7) (Table 2). In addition, mean levels of serum TMEM166 were significantly lower in the control group (12.54 ± 3.74) than in patients with stroke after CEA (38.01 ± 10.01, p<0.05) (Fig. 1I). These results indicated that TMEM166 level correlated positively with the degree of cerebral ischemic injury. Additionally, the levels of serum IL-6 and CRP in stroke patients after CEA were 3.08 ± 0.78 pg/mL and 3.09 ± 1.38 ng/mL respectively, which were significantly higher than those in the control group (1.02 ± 0.27 pg/mL, 1.64 ± 0.6 ng/mL, respectively) (Fig. 1I).

The association between TMEM166 and inflammatory factors in patients was analyzed by Pearson correlation, and the results indicated that the expression of TMEM166 was positively correlated with IL-6 and CPR (r = 0.6, 0.374, respectively, p<0.01) (Supplementary Table 1).

**Figure 3.**
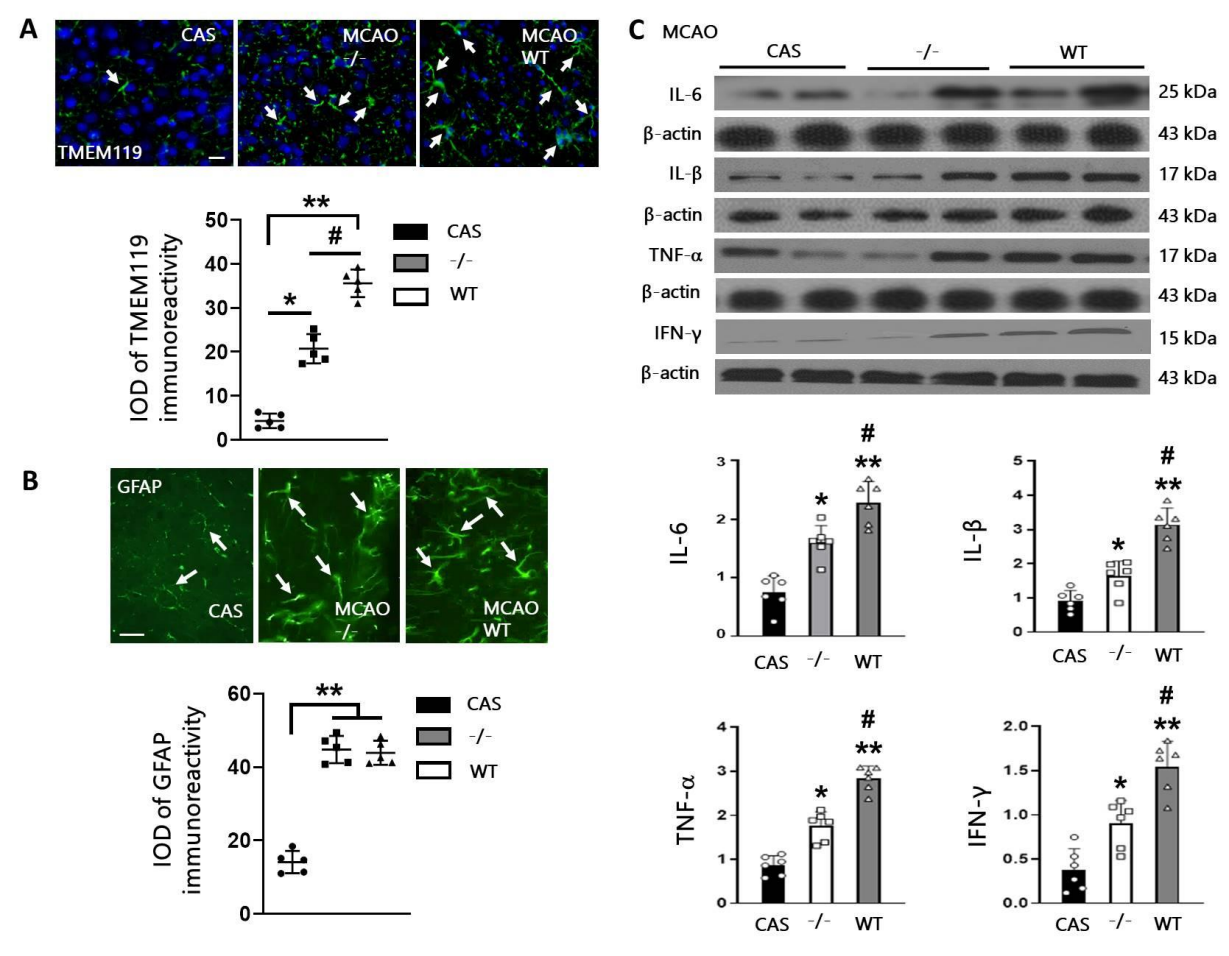
**Increased activation of microglia and high levels of cytokines 24 hours after ischemic stroke secondary to CAS**. (**A-B**), Representative image of TMEM119 and GFAP (green) and the relative IOD immunoreactivity in the cortex in three groups. Brain sections were double-stained with DAPI (blue). Arrows point at TMEM119 or GFAP immunoreactive cells. IOD, integrated optical density. Scale bar = 50 μm. n = 5 per group. (**C**), The expression of cytokines (IL-6, IL-β, TNF-, IFN-γ) was examined by western blot. n = 6 per group. Data are presented as mean ± SD (SD). *P < 0.05, **P < 0.01 compared with the CAS group, ^#^*P* < 0.05 compared with MCAO+TMEM166^-/-^ group, one-way ANOVA with Holm-Sidak test.

### Generation of TMEM166-deficient mice

We bred TMEM166flox/flox mice (Supplementary Fig. 1A) with transgenic Nestin-cre mice to generate TMEM166^-/-^ (flox/flox:Alb-Cre) mice, which exhibited no spontaneous phenotype compared to age- and weight-matched TMEM166^+/+^ (flox/flox) mice. Genotyping of TMEM166^+/+^ and TMEM166^-/-^ mice was confirmed by PCR analysis of genomic DNA extracted from tail samples (Supplementary Fig. 1B). As previously reported [15], quantitative RT-PCR (Supplementary Fig. 1C) and western blot (Supplementary Fig. 1D) analyses demonstrated that TMEM166 was widely expressed in the mouse cerebral cortex, hippocampus, and cerebellum. To further investigate the physiological role of TMEM166 in stroke after CEA, we generated mice carrying the floxed TMEM166 allele (TMEM166flox), which contained two LoxP sites flanking exon 3 of the TMEM166 gene and a neo box.

### TMEM166 knockout decreased the expression of TMEM119 but not GFAP in ischemic stroke secondary to CAS

To investigate the expression of TMEM166 in the brain following ischemic injury, we utilized a CAS model. To verify the success of CAS induction, we measured the CBF of mice in each group and observed a decrease in blood flow after experimental CAS. CBF did not change in the sham group but decreased rapidly after CAS in the wild-type and TMEM166^-/-^ groups, gradually recovering over time, however, remained lower than preoperative levels (Fig. 2A).

To further evaluate if TMEM166 is involved in ischemic stroke, we utilized an MCAO model one month after CAS induction (hereafter referred to as “MCAO”). We used TTC staining (Fig. 2B-C) and MRI scanning (Fig. 2D) to observe cerebral infarction in each group 24 hours after MCAO. The results showed that no cerebral infarction occurred in the CAS group. Brain infarction in the MCAO+TMEM166^-/-^ group was significantly lower than in the MCAO+WT group, but higher than that in the CAS group.

Western blot analysis showed that TMEM166 expression in the MCAO+WT group increased compared to that in the CAS group, while it significantly decreased in TMEM166-deficient mice (Fig. 2E-F). GFAP and TMEM119 [16] were used as specific markers of astrocytes and microglia, respectively. Changes in TMEM119 and GFAP were examined by western blot (Fig. 2G-J) and immunofluorescence (Fig. 3A, B). Both markers increased after MCAO compared to the CAS group. This data suggested that TMEM166 knockout resulted in decreased expression of TMEM119 but not GFAP compared to the wild-type group, which indicates that TMEM166-induced neurological impairment may be related to microglial activation.

### The inflammatory response was induced by microglia in ischemic stroke secondary to CAS

The levels of pro-inflammatory factors, including IL-6, IL-1β, TNF-α, and IFN-γ, which are associated with microglia activation [17], were examined by western blot. At 24 hours after surgery, all these cytokines secreted by microglia in ischemic stroke were increased compared with the CAS group. In contrast, the inflammatory response in MCAO+WT mice was significantly stronger than in MCAO+TMEM166^-/-^ mice, indicating that microglial cells were activated during this process, and knocking out TMEM166 likely inhibited their activation (Fig. 3C).

### The inflammatory responses further promoted the expression of TMEM166

**Figure 4.**
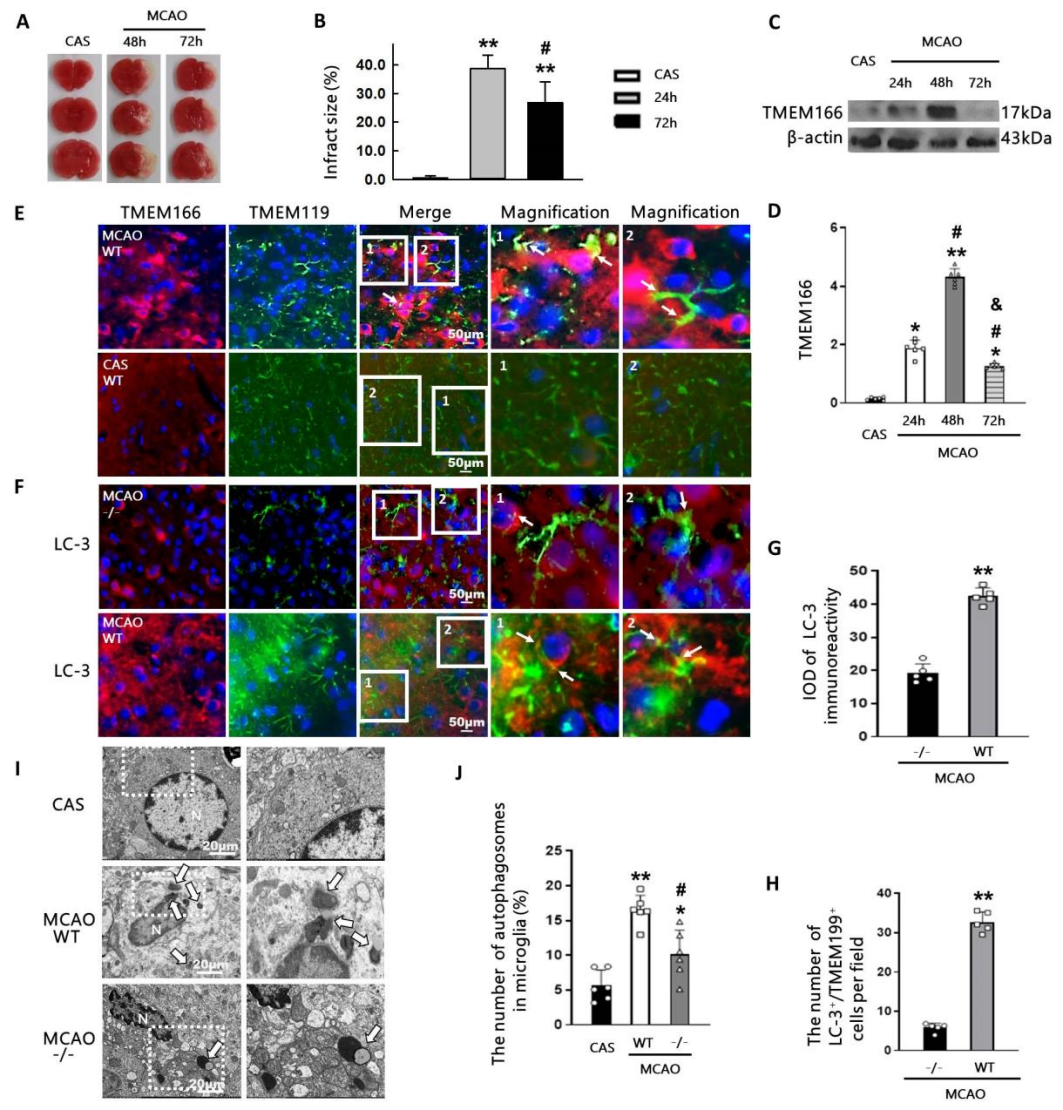
**Inflammatory responses further promoted the expression of TMEM166 and induced microglia autophagy**. (**A-B**), Representative images of TTC stained sections and the quantification of infarct volume at different time points 48 hours after ischemic stroke. n = 8 per group. **P < 0.01 compared with the CAS group, ^#^*P* < 0.05 compared with 24h group. One-way ANOVA with Holm-Sidak test. (**C-D**), The expression of TMEM166 was examined by western blot. The protein level in the CAS group was normalized as 1. n = 6 per group. *P < 0.05, **P < 0.01 compared with the CAS group, ^#^*P* < 0.05 compared with 24h group, &*P* < 0.05 compared with 48h group. Two-way ANOVA with Holm-Sidak test. (**E-F**), Colocalization of TMEM166 (red), LC-3 (red) and TMEM119 (green) in the cortex of brain sections. Rectangles labeled with 1 and 2 were enlarged respectively of the immunofluorescent staining images. Scale bar = 50 μm. (**G-H**), Quantification of LC-3 immunofluorescence intensity, LC-3 and TMEM119 double staining positive cells using ImageJ software in two groups. n = 5 per group. **P < 0.01 compared with MCAO+TMEM166^-/-^ group, t test. (**I**), Representative electron micrograph images of autophagosomes (arrow show) in the cytoplasm of microglia. The images on the right are the corresponding enlarged view of the white box in the left pictures. N: nucleus; scale bar: 20 μm. n = 6 per group. (**J**), Quantification of the autophagosomes in microglia. *P < 0.05, **P < 0.01 compared with the CAS group, ^#^*P* < 0.05 compared with MCAO+WT group, One-way ANOVA with Holm-Sidak test.

The peak of cerebral infarct size occurred 48 hours after ischemic stroke (Fig. 4A, B), coinciding with the highest expression of TMEM166 at the same time point (Fig. 4C, D), indicating a strong association between TMEM166 levels and severe brain ischemia. The increased brain damage may be due to neuroinflammation associated with microglia activation [17]. As shown in Fig. 4E, the degree of microglia activation at 48 hours after cerebral ischemia was significantly stronger than that at 24 hours (Fig. 3A), suggesting a crucial role of TMEM166 at 48 hours post-injury.

### TMEM166-induced microglia autophagy

We observed the occurrence of microglial autophagy 48 hours after ischemic stroke. Immunofluorescence staining showed increased accumulation of TMEM166 and LC-3 (a marker of autophagy) positive aggregates in microglia cells of wild-type mice (Fig. 4E-H). In contrast, the fluorescence intensity of LC-3 was particularly weak in TMEM166^-/-^ C57BL/6 mice, indicating that microglia autophagy was likely regulated by TMEM166. Most interestingly, TMEM166 was also expressed in neurons and endothelial cells in this condition (Supplementary Fig. 2). However, co-staining of TMEM166 and LC-3 was only clearly identified in neurons (Supplementary Fig. 3).

To further investigate the role of TMEM166 in the process of microglial autophagy, we observed the number of autophagosomes in the cytoplasm of microglial cells in MCAO+WT and MCAO+TMEM166^-/-^ groups using transmission electron microscopy (Fig. 4I, J). We did not find autophagosomes in the microglial cells of the CAS group. There was an increase in autophagosomes in WT mice. However, TMEM166 deletion inhibited the number of autophagosomes in mice with MACO injury, indicating that microglial autophagy may be at least partly mediated by TMEM166.

**Figure 5.**
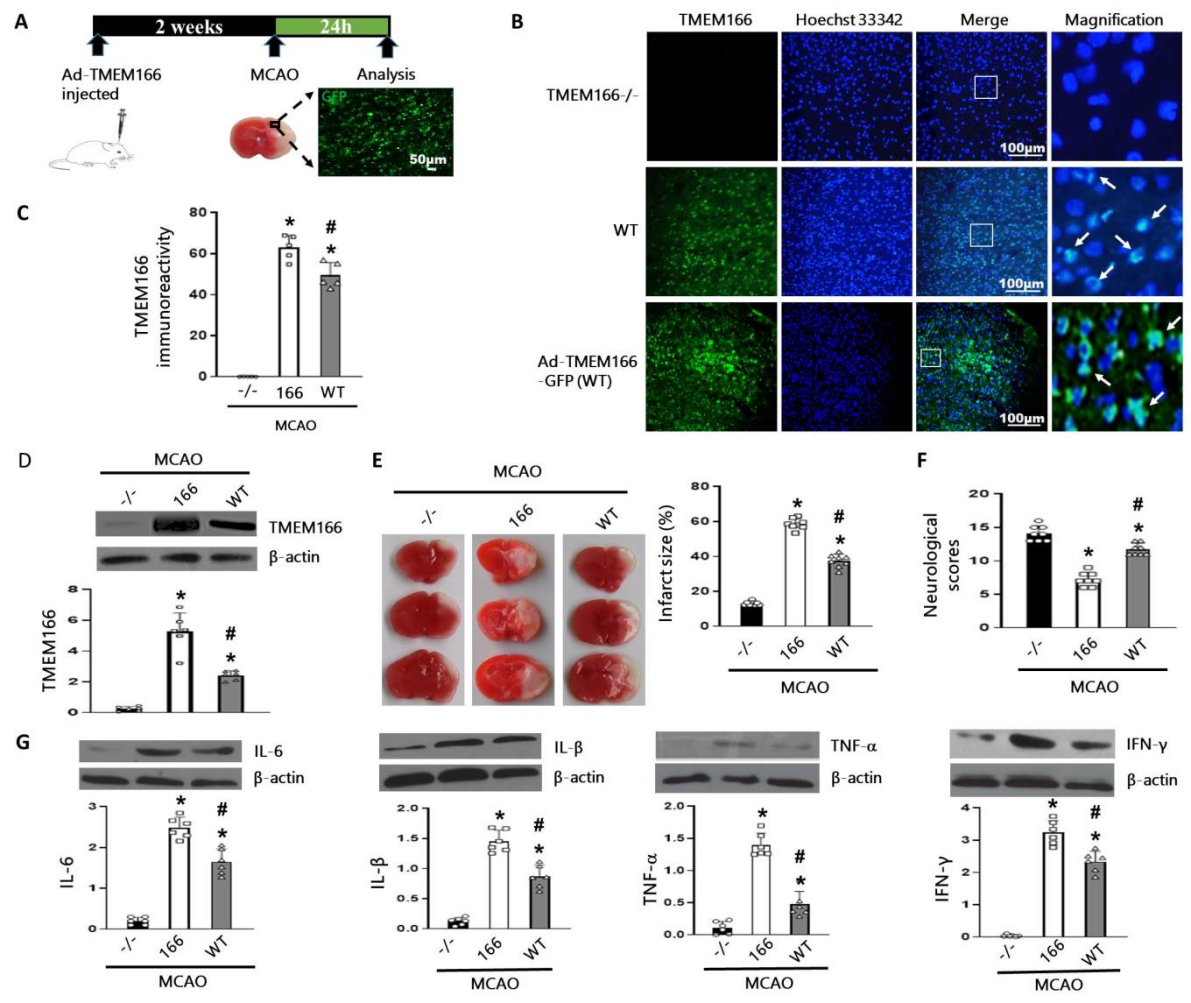
**TMEM166 transfection activated further inflammatory responses and aggravated brain damage**. (**A**), Experimental design and animal treatment. (**B-C**), Representative immunofluorescent images and the immunofluorescence intensity quantification of TMEM166 in the cortex of the mice after MCAO. Scale bar: 100 μm. n = 5 per group. (**D**), Western blot and quantification of TMEM166 in MCAO+TMEM166^-/-^, MCAO+Ad-TMEM166 and MCAO+WT groups. n = 6 per group. (**E-F**), Representative images of TTC stained sections and the neurological scores in different groups. n = 8 per group. (**G**), Western blot and quantification of IL-6, IL-β, TNF-α and IFN-γ in each group. Data are presented as mean ± SD (SD). n = 6 per group. *P < 0.05 compared with the MCAO+TMEM166^-/-^ group, ^#^*P* < 0.05 compared with MCAO+Ad-TMEM166 group, one-way ANOVA with Holm-Sidak test.

### TMEM166 transfection aggravated inflammatory responses and ischemic brain damage

In order to further investigate the role of TMEM166 in this process, Ad5-TMEM166-GFP lentivirus was microinjected into the right lateral cerebral ventricle of mice 2 weeks before MCAO (Fig. 5A) and BV-2 microglial cells. The transfection efficiency was confirmed by immunofluorescence assay and western blot (Fig. 5B-D). Compared to the TMEM166^-/-^ group in mice and the control group in microglial cells, TMEM166 expression in the ischemic brain tissue of the WT group and OGD treatment group increased significantly. As expected, the Ad5-TMEM166 group showed the highest increase in TMEM166 expression both *in vivo* (Fig. 5B-D) and *in vitro* studies (Fig. S4A). Additionally, changes in levels of IL-6, IL-1β, TNF-α, and IFN-γ after TMEM166 transfection were consistent with the level of TMEM166 (Fig. 5G, S4B-E). TTC staining and neurological scores indicated that TMEM166 transfection exacerbated the ischemic brain injury, which was greatly improved by TMEM166 knockout (Fig. 5E, F).

**Figure 6.**
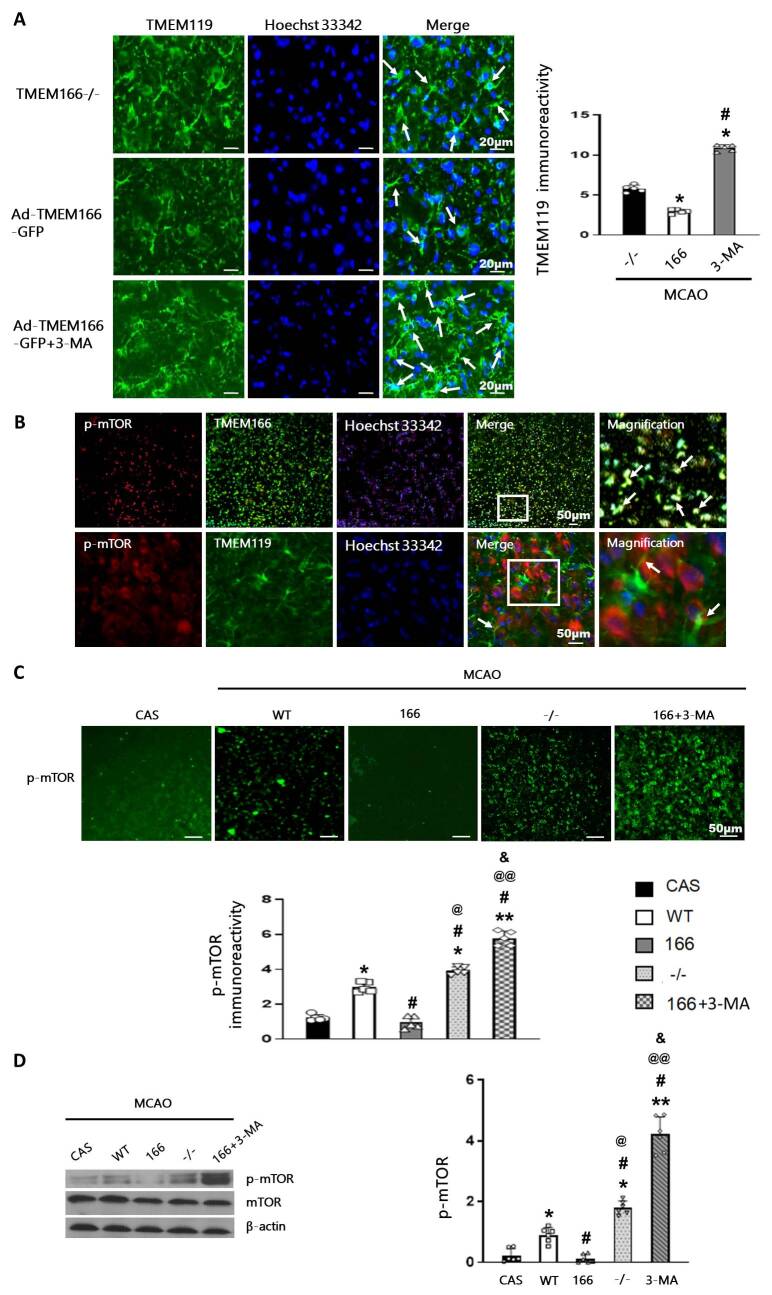
**TMEM166-induced autophagy of microglia was mediated by the mTOR pathway**. (**A**), Representative image of TMEM119 (green) and the relative IOD immunoreactivity in the cortex. n = 5 per group. *P < 0.05 compared with the MCAO+TMEM166^-/-^ group, ^#^*P* < 0.05 compared with MCAO+Ad-TMEM166 group, one-way ANOVA with Holm-Sidak test. (**B**), Colocalization of p-mTOR/TMEM166 and p-mTOR/ TMEM119 in the cortex of mice. n = 3 per group. Red, p-mTOR; Green, TMEM166 or TMEM119; Blue, DAPI. Scale bar: 50 μm. (**C**), Representative immunofluorescent images and the immunofluorescence intensity quantification of p-mTOR in the cortex of the mice after MCAO. N = 5 per group. *P < 0.05, **P < 0.01 compared with the CAS group, ^#^*P* < 0.05 compared with MCAO+WT group, @*P* < 0.05, @@*P* < 0.01 compared with MCAO+ TMEM166 group, &*P* < 0.05 compared with MCAO+TMEM166^-/-^ group, two-way ANOVA with Holm-Sidak test. Scale bar: 50 μm. (**D**), Western blot and quantification of p-mTOR and mTOR in CAS, MCAO+WT, MCAO+TMEM166, MCAO+ TMEM166^-/-^, and MCAO+ TMEM166+3-MA groups. N = 6 per group. *P < 0.05, **P < 0.01 compared with the CAS group, ^#^*P* < 0.05 compared with MCAO+WT group, @*P* < 0.05, @@*P* < 0.01 compared with MCAO+ TMEM166 group, &*P* < 0.05 compared with MCAO+TMEM166^-/-^ group, two-way ANOVA with Holm-Sidak test.

### TMEM166- induced microglia autophagy was mediated by the mTOR pathway

Our findings have shown that despite the knockout of TMEM166, TMEM119 was still highly expressed. However, the number of TMEM119-positive microglia cells decreased significantly after transfection with TMEM166, and this reduction was reversed after treatment with the autophagy inhibitor 3-MA (Fig. 6A). The decrease in the number of microglia cells after transfection with Ad5-TMEM166 suggests that autophagic cell death may have occurred.

Autophagy is initiated when mTOR, the major autophagy suppressor, is inactivated by phosphorylation [18-19]. To investigate the mechanism underlying TMEM166-mediated autophagy in microglia, we examined the expression of phospho-mTOR (p-mTOR) and treated mice with the autophagy inhibitor 3-MA, 30 mg/kg, three times a week for two weeks [20] via intraperitoneal injection before MCAO.

We observed that p-mTOR was expressed in microglia cells in the mouse cerebral cortex and co-localized with TMEM166 (Fig. 6B). Microglial autophagy was significantly activated after transfection with Ad5-TMEM166. The expression of p-mTOR in mice transfected with Ad5-TMEM166 was significantly inhibited, while 3-MA treatment increased its expression. Moreover, TMEM166 knockout mice had more p-mTOR-positive cells compared to the MCAO+WT group (Fig. 6C). Western blot analysis confirmed these results. Compared to the CAS group, the level of p-mTOR in the MCAO+WT group increased, and its expression decreased after Ad5-TMEM166 transfection, while the protein level of p-mTOR significantly increased after the knockout of TMEM166. Consistent with the results of immunofluorescence, the expression of p-mTOR in the 3-MA treatment group was the highest amongst all groups (Fig. 6D).

## DISCUSSION

Despite significant advancements in the surgical management of CAS, postoperative stroke incidence remains high, ranging from 2% to 20% [13, 21]. This can have devastating consequences, as survivors often suffer from serious complications that significantly impact their quality of life. Recently, there has been increasing interest in identifying molecular biomarkers that could predict postoperative stroke. The gene TMEM166, which is associated with cellular survival and autophagy, is being studied to determine if it is related to postoperative stroke after CEA. Previous studies have shown that downregulation of TMEM166 inhibits autophagy in HeLa cells [22], and that TMEM166-induced neuronal autophagy is involved in brain injury during cerebral ischemia-reperfusion injury in rats [2].

Abnormal migration and proliferation of vascular smooth muscle cells (VSMCs) are key triggers of atherosclerosis and stenosis [23]. Inflammation has been shown to contribute to the abnormal migration of VSMCs and the formation of atherosclerotic plaques [24-27], and autophagy has a close association with inflammation [28]. In this study, TMEM166 expression was detected in the inner part of the sclerotic plaque, and the expression was significantly higher than in other segments of the plaque, accompanied by a large amount of enrichment of inflammatory mediators. Furthermore, serum levels of TMEM166 and inflammatory mediators in perioperative stroke patients were significantly higher than in the control group. Correlation analysis showed that the autophagy-related protein TMEM166 had a positive correlation with inflammatory mediators, indicating that inflammation and autophagy may be involved in stenotic carotid arteries and postoperative stroke after CEA.

TMEM166^-/-^ mice were generated and an MCAO model was utilized following CAS injury to test the hypothesis. The successful conduction of the model was evaluated by measuring CBF. The wild-type mice subjected to MCAO injury after CAS showed poor neurological function compared with TMEM166^-/-^ mice. TMEM166 expression was slightly elevated in CAS mice, whereas the expression was significantly higher in MCAO animals after CAS, indicating a positive correlation with the degree of ischemic brain injury. These results are consistent with the clinical studies in CEA patients.

Activation of microglia is a key factor in ischemic brain injury [29-30] and is involved in the development of cerebral hypoperfusion by increasing the expression of pro-inflammatory cytokines [31]. TMEM166 deficiency can protect mice from such damage by suppressing inflammatory cytokine expression. The current study demonstrated the activation of astrocytes and microglia 24 hours after cerebral ischemia and found that knocking out TMEM166 significantly inhibited the activation of TMEM119-positive microglia, but not GFAP-positive astrocytes. Inflammatory responses further promoted the expression of TMEM166, which induced microglia autophagy.

Qin et al. previously detected microglial autophagy after ischemic white matter injury [32]. Autophagy is one of the mechanisms used by microglia to clear damaged cells and debris within the injured brain. Therefore, we hypothesized that the induction of autophagy in microglia may be a key mechanism for the clearance of damaged cells and debris in the brain after ischemic stroke following CAS. In our current study, we found that knocking down TMEM166 inhibited the expression of the autophagy-related protein LC-3 in microglia and reduced the number of autophagosomes, suggesting that TMEM166 can induce autophagy in microglia after cerebral ischemia. Additionally, we observed that p-mTOR was expressed in microglia and co-expressed with TMEM166, leading us to speculate that TMEM166-induced microglial autophagy might be mediated by mTOR. Mammalian autophagy is initiated by the phosphorylation inactivation of mTOR [33]. Previous studies have suggested that mild autophagy can alleviate nervous system damage, while excessive autophagy is detrimental [34-35]. To explore the role of TMEM166-induced autophagy in microglia, we transfected mice with Ad5-TMEM166. We found that TMEM166 transfection exacerbated the inflammatory response and aggravated brain injury, accompanied by a sharp decrease in the number of microglia and the expression of p-mTOR. We also found that the reduction of microglia was reversed by administration of 3-MA, while knockout of TMEM166 upregulated the expression of p-mTOR. Our findings suggest that excessive autophagy of microglia occurred during this process and that the latter might be mediated by p-mTOR.

**Figure 7.**
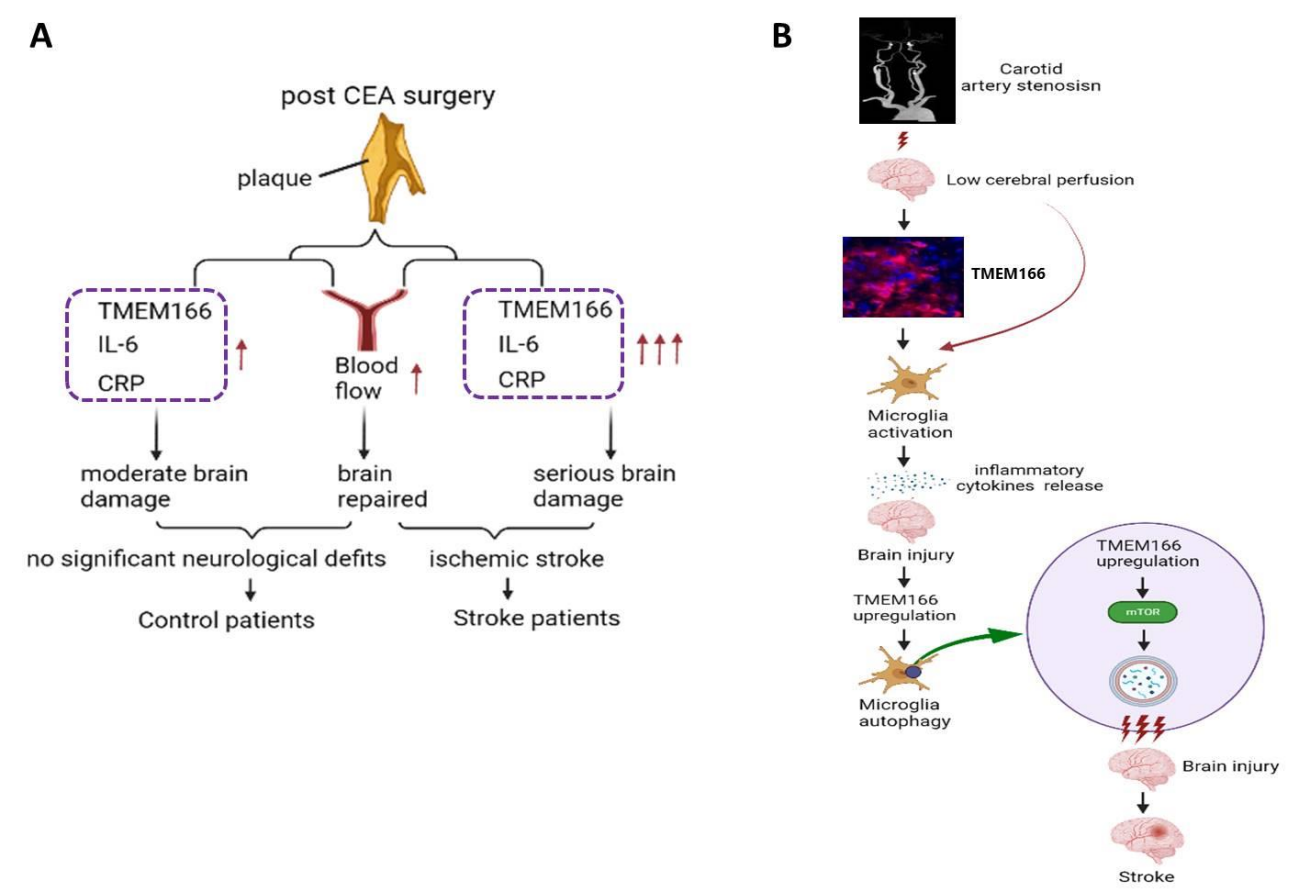
**The proposed pathway by which TMEM166 regulates stroke progression**. (**A**), Carotid endarterectomy on the one hand increases the cerebral blood flow and improves brain function, on the other hand caused TMEM166, IL-6 and CRP upregulation, which may be closely related to brain damage. In control patients, TMEM166, IL-6 and CRP suffer a moderate increase, while they increase remarkably in stroke patients. (**B**), Microglia autophagy mediates by TMEM166/mTOR pathway. Carotid artery stenosis causes low cerebral perfusion and the subsequent TMEM166 upregulation and microglia activation, which results in inflammatory cytokines release and a mild brain injury. The brain injury further promotes the over expression of TMEM166 and the mass activation of its downstream mTOR, leading to an enhanced autophagy of microglia and an ischemic stroke.

In this study, we present novel evidence demonstrating that TMEM166 plays a critical role in stroke after CAS and may be associated with the extent of perioperative brain injury following cerebral ischemia. Specifically, our results indicate that TMEM166 can prompt ischemic brain injury by activating microglial activation, inflammatory response, and microglial autophagy (Fig. 7). Our findings suggest that TMEM166 may serve as a reliable prognostic marker for the early detection of postoperative stroke and improve clinical outcomes. Further preclinical studies are necessary to clarify the role and mechanism of TMEM166 in postoperative stroke after CEA.

## Supplementary Materials

The Supplementary data can be found online at: www.aginganddisease.org/EN/10.14336/AD.2023.0803.


