# Supplementary material for: Microglia Autophagy Mediated by TMEM166 Promotes Ischemic Stroke Secondary to Carotid Artery Stenosis
Source: Aging Dis. 2024 May 7;15(3):1416–31. doi: 10.14336/AD.2023.0803 (PMC11081158; doi:10.14336/AD.2023.0803)
Supplement: Supplementary file 1 [file AD-15-3-1416-s.pdf]

SUPPLEMENTARY DATA

Microglia Autophagy Mediated by TMEM166 Promotes Ischemic Stroke Secondary to Carotid Artery Stenosis

Li Li, Paul R. Krafft, Na Zeng, Ranran Duan, Xiang Qi, Anwen Shao, Fushan Xue, John H. Zhang

© 2023. Li L et al. Published online at http://www.aginganddisease.org/EN/10.14336/AD.2023.0803

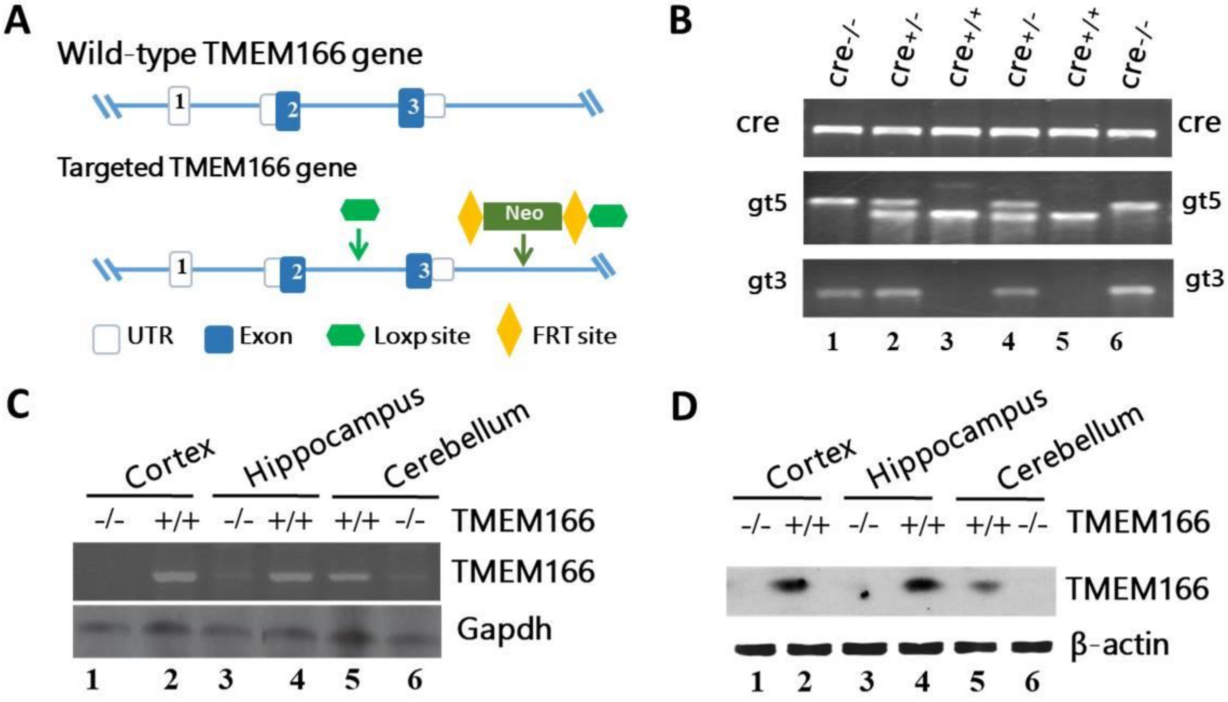

Supplementary Figure 1. TMEM166$^{-/-}$ mice generation. (**A**), Scheme to generate TMEM166 conditional knockout mice. (**B**), PCR was carried out using genomic DNA extracts from mouse tails venous. (**C**) and (**D**), Brain cortex, hippocampus, and cerebellum were obtained from TMEM166$^{+/+}$ mice and TMEM166$^{-/-}$ mice.

© 2023. Li L et al. Published online at http://www.aginganddisease.org/EN/10.14336/AD.2023.0803

SUPPLEMENTARY DATA

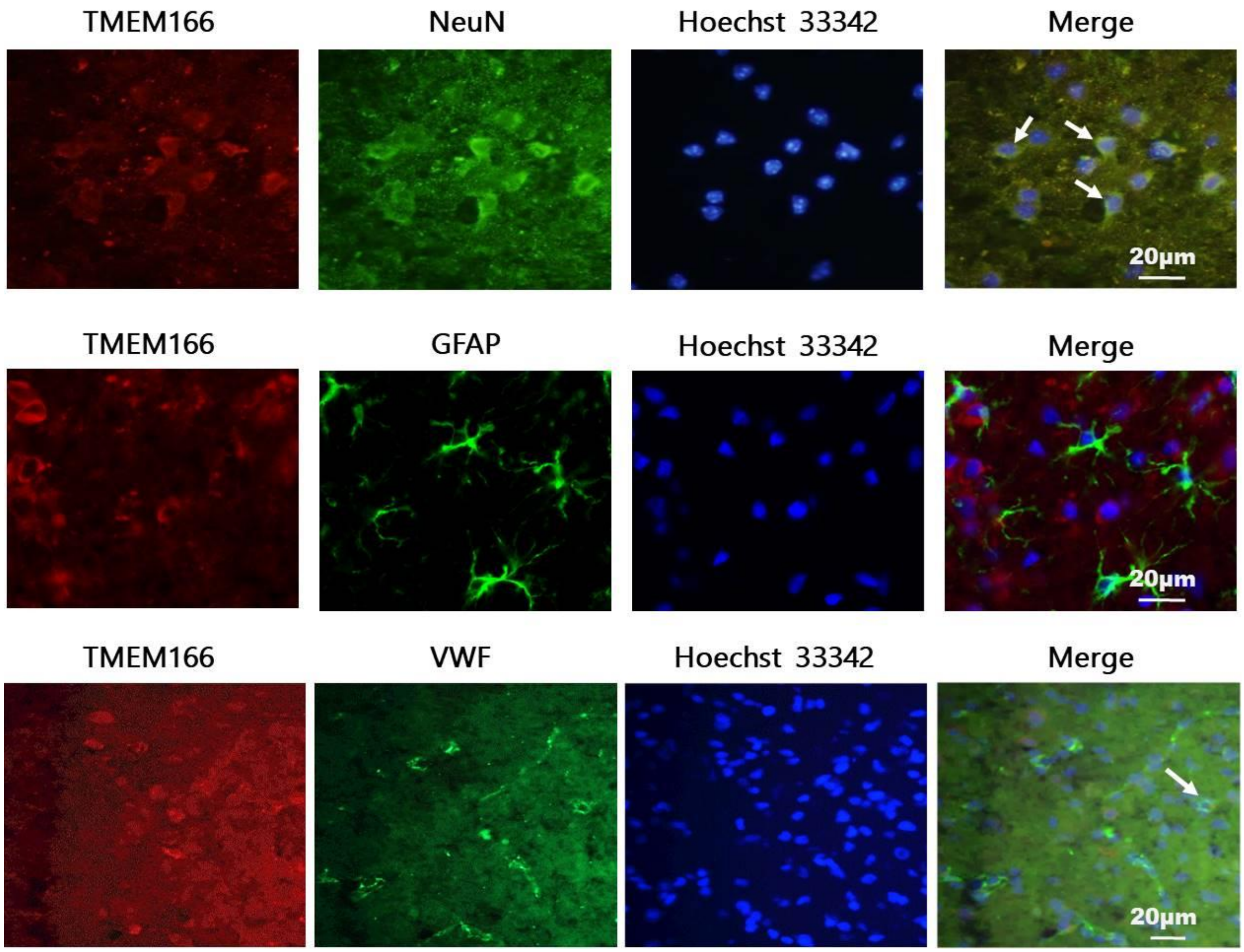

Supplementary Figure 2. TMEM166, NeuN, GFAP, TMEM119, and VWF expression. The immunofluorescence stainings of TMEM166, NeuN (neuron marker), GFAP (astrocyte marker), TMEM119 (microglia marker), and VWF (endothelial cell marker) in the brain after ischemic stroke secondary to carotid artery stenosis. Scale bar = 20 µm.

© 2023. Li L et al. Published online at http://www.aginganddisease.org/EN/10.14336/AD.2023.0803

SUPPLEMENTARY DATA

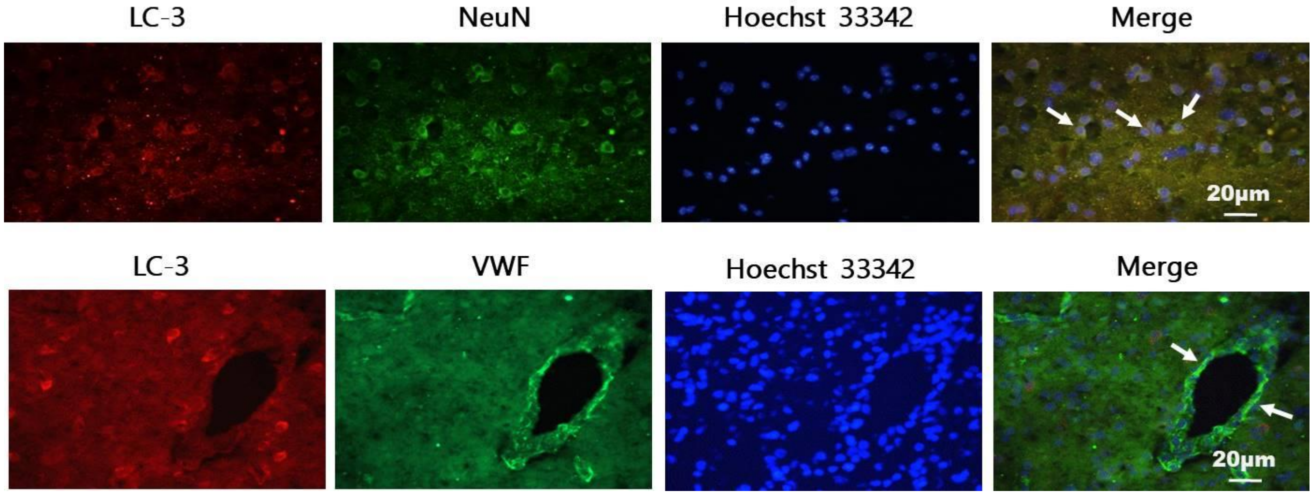

Supplementary Figure 3. LC-3, NeuN, and VWF expression. The immunofluorescence stainings of LC-3, NeuN, and VWF in the brain after ischemic stroke secondary to carotid artery stenosis. Scale bar = 20 μm.

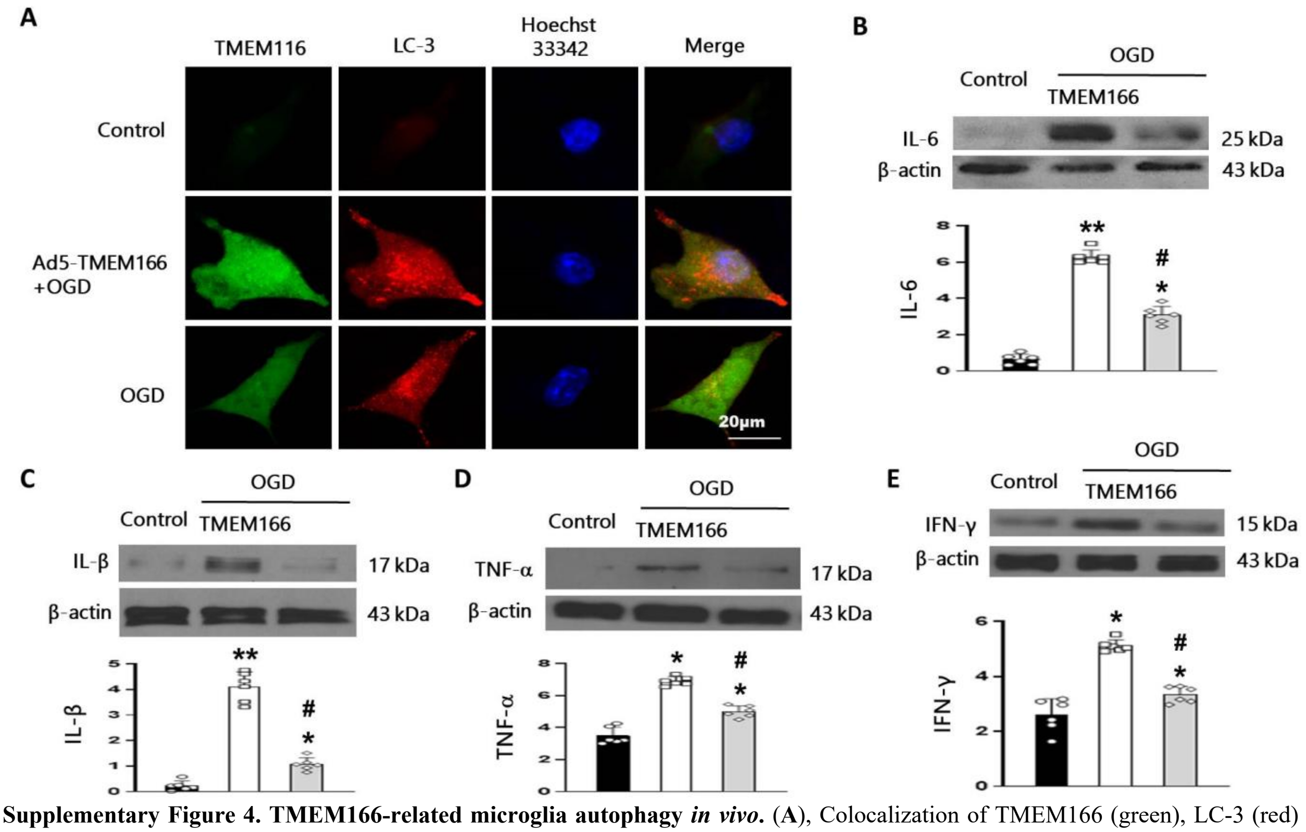

Supplementary Figure 4. TMEM166-related microglia autophagy *in vivo*. (**A**), Colocalization of TMEM166 (green), LC-3 (red) and Hoechst 33342 (blue) in Control, Ad5-TMEM166+OGD and OGD group in BV2 microglial cell. Scale bar = 20 μm. (**B-E**), The expression of cytokines (IL-6, IL-β, TNF-α, IFN-γ) was examined by western blot. n = 6 per group. Data are presented as mean ± SD (SD). **P* < 0.05, ***P* < 0.01 compared with Control group, #*P* < 0.05 compared with Ad5-TMEM166+OGD group, one-way ANOVA with Holm-Sidak test.

© 2023. Li L et al. Published online at http://www.aginganddisease.org/EN/10.14336/AD.2023.0803
